# The impact of decision models on self-perceived quality of life: a study on brain cancer patients

**DOI:** 10.3332/ecancer.2010.187

**Published:** 2010-08-20

**Authors:** C Lucchiari, A Botturi, G Pravettoni

**Affiliations:** 1 Dipartimento di Studi Sociali e Politici, Università degli studi di Milano, Via Conservatorio 7, 20122 Milano, Italy; 2 Italian National Neurological Institute C Besta, Via Celoria 11, 20133 Milan, Italy

## Abstract

Quality of life (QoL) is an increasingly important outcome measure in medicine. Health, in fact, is not only based on functional status but also on psychological and social well being. Since QoL is related to the patient’s perception of their position in life in relation to their goals, expectations, standards and concerns, the way in which the medical context is experienced may be critical. We then hypothesised that self-perceived QoL may be linked to unmet needs in information management and decision involvement. To analyse this hypothesis, we conducted a quantitative study on 84 consecutive patients with a diagnosis of primary high-grade glioma. The functional assessment of cancer therapy-Brain (FACT-Br) scales, the hospital anxiety and depression (HAD) scale and the need evaluation questionnaire (NEQ) questionnaire were used, in order to measure quality-of-life dimension, mood and unmet needs. Patients were classified as having no need (cluster 1), a moderate need (cluster 2) or a high need (cluster 3) to be more involved in the clinical process.

Our data confirmed previous studies in other clinical areas, showing that shared decision might contribute to a better adaptation process to the illness [1]. In fact, patients in cluster 1 showed a significant better self-perceived QoL, despite the lack of clinical differences between clusters. The study showed that patients satisfied with respect to decisional involvement seem to be able to better cope with their disease. Finally, the study suggests the need for a more attuned decision-making process in approaching clinical decisions. Physicians need to better understand patient preferences related to information and decision sharing.

## Introduction

Brain cancers contribute to about 2% of the cancer mortality in men and 1.4% of the mortality in women, and within the age group 15–34 years, they are the third most common cause of death due to cancer. The most common types of brain tumours are glioblastoma multiforme (39% of all brain tumours), a high-grade (grade IV) astrocytic tumour that is almost always debilitating and rapidly fatal (6% survive 2 years) and anaplastyc astrocytoma (grade III). Despite recent advances in diagnosis only modest improvements in survival are evident. However, the number of therapeutic options is increasing, and the importance of quality of life (QoL) is now well stated. In the last 10 years, several studies have investigated QoL issues in brain cancers. In particular, it has been shown that is possible to sustain a good QoL even though the prognosis remains poor. This may be due to the higher tolerability of treatments, but also to the specific attention that medical equips now give to QoL issues [2]. In particular, depression, anxiety and, generally speaking, emotional wellbeing are now common clinical targets. However, the available data are not always in agreement. Some studies, in fact, showed that most patients have pathological levels of depression, while others say the opposite [3–5]. Furthermore, it is not clear which patient characteristics may be important in influencing QoL. Some studies highlighted the role of the lesion site, functional status, physical symptoms, social support, coping strategies and gender [6–10]. However, the data do not seem to be conclusive. Furthermore, few researchers have investigated the relationship between QoL and the patient/physician relationship. In particular, specific needs concerning information and decision sharing are poorly investigated in the domain of brain cancer QoL. In fact, QoL is a multidimensional concept that may be defined as the state of wellbeing that is a composite of two components: the ability to perform everyday activities that reflect physical, psychological and social well being and patient satisfaction with levels of functioning and control of the disease [11]. In this sense, we believe that the way patients experience the clinical setting may play a key role in the adjustment process to the illness and consequently in sustaining QoL.

In the shared decision-making model, the power of control over the decision-making process is entirely entrusted to a patient. The interaction between patients, physicians and others (family members, for instance) is the starting point in the deliberation for the final decision making [12]. So, a patient has to be well informed on aetiology, symptoms, diagnostic methods, prognoses, advantages and eventual risks of different treatments in order to evaluate together with a physician the possible treatment alternatives in a conscientious and critical way. Thus, information is the crucial power for a patient, i.e., it is an essential condition for so-called empowerment.

Consequently, the medical system should be able to be attentive to patient’s requirements and to satisfy their needs [13]. It means that physicians have to be able not only to provide patients with professionalism, respect for beliefs and convictions, but also have to meet their needs on being involved in the treatment details and therapeutic choices.

Generally speaking, shared medical decision-making is supposed to be an ideal model to frame the patient/physician encounter [14]. Though the generalities of this method are largely accepted, the basic shared decision-making principles are not in reality always applied. Furthermore, the real preferences and needs of patients with regard to shared decisions are not systematically considered in clinical settings. Thus, the decision model to be applied depends often on the physician’s attitude and evaluations.

The present study was aimed at evaluating objective indexes of health related quality of life in patients grouped by different shared decision needs. In particular, we wanted to verify the effect of unmet needs in this domain. In fact, we may hypothesise that the mismatch between the decision models used in the clinical setting, and the specific need of a patient to be involved may negatively contribute to self-perceived QoL. The need to have more information and/or to be more involved in medical decisions may interfere with the production of a context of trust and satisfaction with the therapeutic journey, holding back the adjustment process.

## Method

### Subjects

109 consecutive patients were contacted. All the patients admitted were assessed for cognitive status. The first contact with the patient was carried out through the patient’s physician who was considered to be the reference person in determining the general condition of the patient. 10 patients were excluded for serious clinical conditions and 15 declined to participate.

84 consecutive inpatients (mean age = 49.8, range = 26–65) with a histological diagnosis of malignant brain cancer (GBM or grade III Anaplastic Astrocytoma) agreed to take part in the study. All the patients were diagnosed within the three months prior to participating in the study, and they were undergoing chemotherapy and/or radiotherapy.

Once the patient had been introduced by their physician, one specialized researcher explained the methods and purposes of the study. At this point, all privacy and ethical issues were raised, discussed and explained. A written form containing all the procedures, methods and contacts was given to the patient to take away, so as to give him/her the possibility to discuss potential participation also with proxies.

During a second encounter, the patient was asked about their decision, if required further information was given at this point. A standard written informed consent was given to the patient and it’s content explained to the patient; if the patient agreed to participate he signed and returned the form.

All contacted patients were aware that they had been operated on for a primitive cerebral neoplasm, and that they would need other therapies such as chemotherapy and radiotherapy to prevent recurrence. The patient’s physical and functional performance status were assessed through the Karnofsky performance status (KPS); the cognitive state was assessed by the mini-mental state examination (MMSE). To avoid any influence on patients QoL assessment, the interview was conducted by researchers not involved in the primary care.

Follow-up visits were performed three months after the first session, to evaluate the QoL trend among patients with different needs. We chose to apply a second QoL evaluation after a short but salient period of time (three months), to test whether patients with different degrees of needs satisfaction developed different illness adjustment processes. In this period, the patient has had time to adjust to his/her new context following brain cancer diagnosis, and we could evaluate the impact of psychological variables on their QoL. A longer time interval may have allowed for physical deterioration, and so organizational factors could have interfered with our ability to test the hypothesis.

The procedure used was in accordance with the ethical standard of the institutional committee on human experimentation.

Patient characteristics are reported in the following Tables 1 and 2.

## Instruments

### The Karnosky performance status scale

The Karnosky performance status scale is a 100 point rating index widely used by physicians to assess patients’ physical and functional performance abilities. The value range was from 0 (dead) to 100 (no impairment, normal activity).

### The mini-mental state examination

This is a broadly used neuropsychological test used to briefly assess the cognitive status of patients. It is validated for Italian culture and corrected for age and education level. The value range was from 0 meaning the worst and 30 the best score.

### The hospital anxiety and depression scale

The HADS is a self-administered question composed by two scales containing seven items, one for anxiety and one for depression, which should be used as two separate measures of emotional distress.

The scale has been previously validated for Italian culture by Costantini and showed a high internal consistency with Crohnbach’s alpha ranging between 0.83 and 0.85 [15].

This scale evaluates symptoms of anxiety and depression avoiding misattribution due to physical aspects of the illness. The value range was from 0 to 21 for each scale. Cut-off scores were preliminary defined as: normal 0–5, light 6–8, moderate 9–11 and heavy more than 11 either for the anxiety and depression patients [15].

### The functional assessment of cancer therapy—brain

The FACT-Br [16] is a self-administered questionnaire that measures the health-related quality of life. This instrument is mainly used for patients with chronic illnesses.

It is composed by a core questionnaire called the Functional Assessment of Cancer Therapy General (FACT-G) and a specific subscale for brain.

The FACT-G is composed of 27 items and has a five-point Likert scale (0–4). The higher scores correspond to better self-perceived QOL.

The FACT-G is divided in four domains: physical well being (seven items), social/family well being (seven items), emotional well being (six items) and functional wellbeing (seven items). The range of the score is 0–28 except emotional well being which is 0–24. A standardized score exists to transform raw data in a 0–100 scale, 100 corresponding to the best score. The specific brain cancer scale is composed by 19 further concerns items. Each item is based on the same five-point Likert scale and is specific for brain cancer problems. The score range is 0–76. The sum of the four sub-scales of the core instrument and the specific brain scale give the FACT-Br total score is 0–184.

### Need evaluation questionnaire

To evaluate patients’ needs, we used the NEQ [17]. This a questionnaire developed by Tamburini and colleagues [17] at the Cancer Institute of Milan. The questionnaire is made up of 19 items, each one representing a specific need in an affirmative mode (e.g. ’I need psychological support‘). The patient has to cross ‘yes’ or ‘no’.

The first section (information management needs), made up of eight items, was considered in particular (see Table 3).

## Statistical analyses

We classified patients for unmet needs with regard to decision sharing into three clusters (1, 2 and 3 involvement need). In the first cluster, patients that presented complete satisfaction about decision and information management were included. This does not mean that patients in this cluster had higher needs regarding involvement in clinical decisions, simply that their needs were described as being met. In the second cluster, patients were included who reported the need ‘to be more involved in therapeutic decisions’ (item 5 of the NEQ). Lastly, patients in the third cluster presented at least three unmet needs with regard to information sharing on the NEQ.

To verify the hypothesis that unmet needs with regard to clinical decision involvement may affect patients QoL, we performed a series of analysis of variance (ANOVA), using QoL scores as dependent variables. All QoL scores were tested for normality with the Shapiro–Wilk test, showing p-values higher than 0.3 in all cases. Non-parametric tests were used when appropriate.

All the statistical analyses were performed using the SPSS statistical software, version 17.0, and the results were considered statistically significant when the p-value was <0.05.

## Results

Clusters composition is shown in Table 4.

We compared these three clusters for gender, age, education, MMSE and KPS, to verify the homogeneity of clusters. The only significant difference was found in gender distribution among the three clusters (χ^2^ = 6.135, p = 0.018). In fact, cluster 3 is clearly unbalanced since it includes many more men than women. No other effects were found, confirming the absence of significant clinical differences.

The ANOVA test showed no significant difference between general QoL measures (FACT-G: *F*(2,83) = 0.579, p = 0.562; FACT-Br: *F*(2,83) = 1.105; p = 0.379) between the three clusters considered. The Kruskal–Wallis non-parametric test also showed that the three clusters did not differ for depression (χ^2^ = 0.202, p = 0.911) or anxiety symptoms (χ^2^ = 1.242, p = 0.513).

Since general scores comprised physical, social and psychological aspects, we carried out other ANOVA tests on the single FACT scales, using needs cluster as a fixed factor.

The ANOVAs showed significant difference only for two sub-scales: the emotional well being scale [*F* (2, 83) = 4.403, p =0.015, 1-β = 0.775] and the socio/familial scale [*F*(2, 83) = 3.801, p = 0.028, 1-β = 0.812].

Bonferroni *post hoc* analysis showed that patients in cluster 1 reported significantly different values for socio/familial well being with respect to cluster 2 (mean difference = 9.65, p = 0.013) and cluster 3 (mean difference = 9.75, p = 0.014). Similarly, patients in cluster 1 reported different values in the emotional domain with respect to cluster 2 (mean difference = 8.78, p = 0.023) and to cluster 3 (mean difference = 8.15, p = 0.034). As we can see from Table 5, patients in cluster 1 showed better emotional and socio/familial indexes (see Table 5).

Since the subject distribution in the three clusters is unbalanced for gender, we performed further two way ANOVAs on QoL scores, using needs clusters as fixed factors and also gender as a fixed factor. The results showed that the needs cluster main effect were significant for emotional well-being [*F*(2, 83) = 3.94, p =0.023, 1-β = 0.755] as well as the interaction gender per cluster [*F*(2, 83) = 4.813, p = 0.010, 1-β= 0.810]. In particular, as shown in Table 6, women reported a higher value than men in cluster 3. No other effect was significant (p > 0.05 in all cases).

Three months after the first assessment, we conducted a follow-up session to evaluate the QoL trend among the three clusters. Eighty-one patients participated in the follow-up. A mixed ANOVA, with needs (three levels) as between factor, and time (two levels) as within factor, was performed using FACT-Br as the dependent variable. No effect was found to be statistically significant (p >0 .10 in all cases). Since FACT-Br is a complex measure, we performed further ANOVA with needs (three levels) as between factor, and time (two levels) as within factor, using FACT sub-scales as dependent variables.

The results indicated a significant interaction of needs and time for emotional [*F* (2, 80) = 11.312, p = 0.003, 1-β = 0.829] and social sub-scales [*F*(2,80) = 7.523, p = 0.011, 1-β= 0.788]. In particular, patients in clusters 2 and 3 showed a significant increase in both emotional and social well being, while patients in cluster 1 showed no particular differences.

## Conclusion

A high-grade glioma is often a fatal diagnosis and the hope of living longer than five years overall for GBM, the most frequent histology, is poor. Although the patient is ‘terminal’ there are different therapeutic options which have been shown to be compatible with an acceptable quality of life [6]. Some studies have also addressed the possibility of further improving this situation through specific intervention (i.e. [18]). In this framework, it is vital to analyse all the conditions that may support psychological adjustment to this difficult journey. Different studies have shown the importance of treatment tolerability [8], symptoms relief [3], and psychological wellness [19, 20]. Furthermore, we know that subjective expectations are very important for the patient’s perception of their QoL [21, 22]. However, it is not clear how a patient builds their expectations and how they manage to fit the contextual demands of a severe diagnosis such as a brain cancer. Understanding unmet needs with regard to decisions involvement could represent a privileged way of understanding patients’ expectations. In particular, our study underlines the importance of evaluating patients’ needs so as to attune the physicians’ behaviour with the patients’ requirement.

Involving patients in decision making is becoming an important clinical task. However, it is essential to differentiate patients with regard to preferences and needs .We believe that a brief but systematic screening of patient characteristics is required in medical settings where important choices have to be made.

The present research confirms previous studies in other clinical areas, showing that decision sharing might contribute to a better adaptation process to the illness [1].

However, to our knowledge this is the first study that addresses the possible impact that an attuned decision model may have within the context of primary brain tumours. Our study strongly suggests the need for a more attuned decision-making process for approaching clinical decisions, even though aggressive therapies and a poor prognosis make it a difficult context for physicians and patients to discuss. The physician/patient encounter could be structured in order to meet reciprocal needs, improving compliance and satisfaction. Our study suggests that within a similar encounter a shared decision model should be systematically applied.

The present research has a number of methodological constraints that limit generalizations. The sample was relatively small, although comparable with other studies, as high-grade gliomas are a relatively infrequent pathology. Overall, most of our subjects had an acceptable KPS index, indicating the absence of particularly disabling symptoms and deficits. In patients with a KPS index lower than 70, or when symptoms are not well controlled by therapies (such as seizure), the QoL is strongly affected by physical conditions. Also psychological distress may contribute to a poor QoL, for instance in patients with a high level of anxiety or depression [23]. Our sample presented a low to moderate level of psychological distress, and this could limit the generalization of our data. However, since many patients with high-grade gliomas may live from some months to some years, with a substantially stable physical situation, we believe that it is important to identify how best to sustain the best QoL. In this framework, our study addressed the importance of considering the adequacy of information management and patient involvement. The study also addresses the need to provide physicians with standardized tools to evaluate this domain of the patient experience, often left to the qualitative assessment of the medical equipment.

## Figures and Tables

**Table 1:**
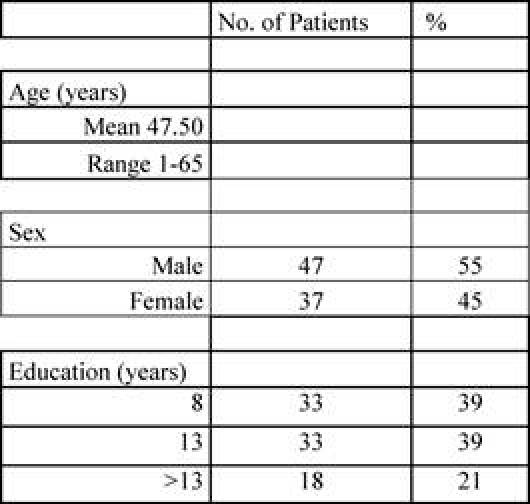
Patients’ characteristics

**Table 2:**
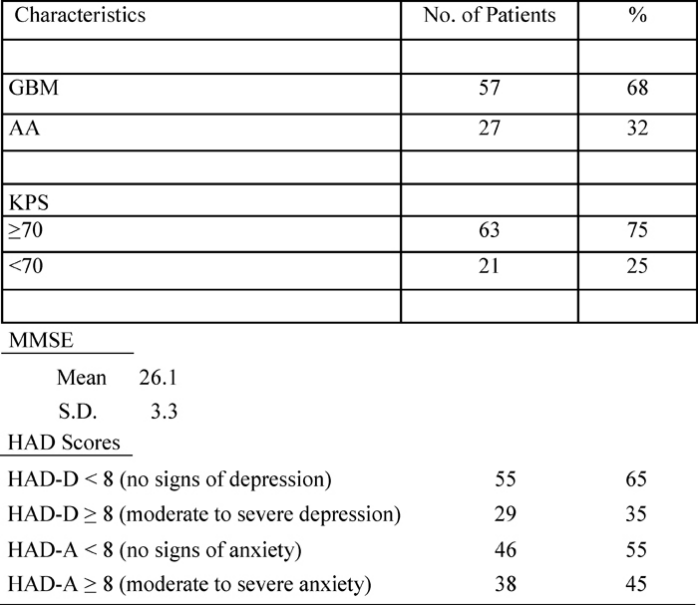
Clinical characteristics

GBM = Glioblastoma multiforme; AA = anaplastic astrocytoma; KPS = Karnofsky performance status; MMSE = mini-mental state examination; HAD = hospital anxiety and depression; HAD-D = hospital anxiety and depression-D: HAD-A = hospital anxiety and depression-A

**Table 3:**
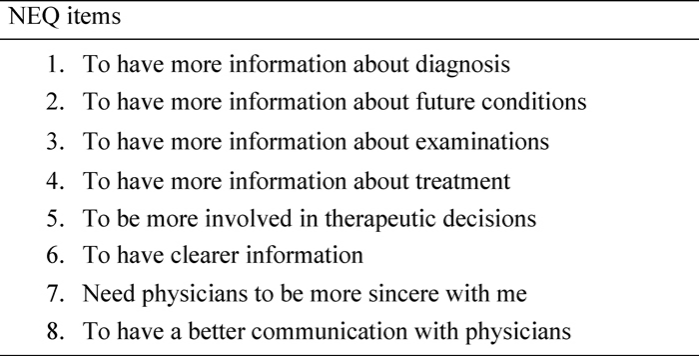
Need evaluation questionnaire (NEQ). The table shows the first eight items of the NEQ, that is the information management session.

**Table 4:**
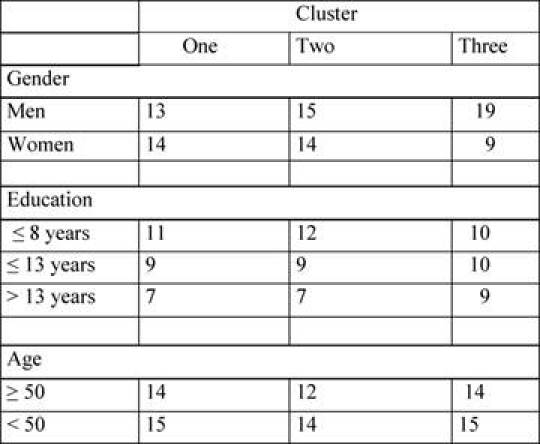
Subjects characteristics distributions among needs clusters

**Table 5:**
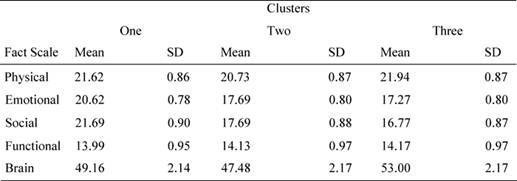
Mean values and standard deviation for FACT sub-scales

**Table 6:**
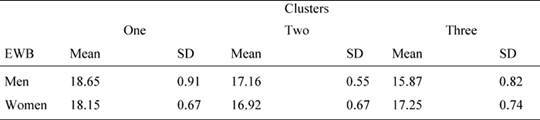
Mean values and standard deviations of the FACT emotional well-being (EWB) scale

## References

[1] Hubbard G, Kidd L, Donaghy E (2008). Preferences for involvement in treatment decision making of patients with cancer: A review of the literature. Eur J Oncol Nurs.

[2] Efficace F, Bottomley A (2003). Assessing HRQOL: a neglected issue for high-grade glioma. Lancet Oncol.

[3] Giovagnoli AR (1999). Quality of life in patients with stable disease after surgery, radiotherapy, and chemotherapy for malignant brain tumour. J Neurol Neurosurg Psychiatr.

[4] Pringle AM, Taylor R, Whittle IR (1999). Anxiety and depression in patients with an intracranial neoplams before surgery. Br J Neurosurg.

[5] Brown PD, Ballman KV, Rummans TA, Maurer MJ, Sloan JA, Boeve BF (2006). Prospective study of quality of life in adults with newly diagnosed high-grade gliomas. J Neurooncol.

[6] Budrukkar A, Jaliali R, Dutta D, Sarin R, Devlekar R, Parab S, Kakde A (2009). Prospective assessment of quality of life in adult patients with primary brain tumours in routine neurooncology practice. J Neurooncol.

[7] Wyszko E, Rolle K, Nowak S, Zukiel R, Nowak M, Pierstrzeniewicz R, Gawronska I, Barciszewska MZ, Barciszewski J (2008). A Multivariate analysis of patients with brain tumors treated with ATN-RNA. Acta Pol Pharm.

[8] Bampoe J, Laperriere N, Pintilie M, Glen J, Micallef J, Bernstein M (2000). Quality of life in patients with glioblastoma multiforme participating in a randomised study of brachytherapy as a boost treatment. J Neurosurg.

[9] Baider L, De-Nour P, Kaplan A (1997). Psychological Distress and Intrusive Thoughts in Cancer Patients. J Nerv Ment Dis.

[10] Jenkins R, Pargament K (1995). Religion and spirituality as resources for coping with cancer. J Psychosoc Oncol.

[11] Gilbert MT, Meyers CA (2000). Issues in assessing and interpreting quality of life in patients with malignant glioma. Semin Oncol.

[12] Gaston CM, Mitchell G (2005). Information giving and decision-making in patients with advanced cancer: a system review. Soc Sci Med.

[13] Kaplan RM, Frosch DL (2005). Decision Making in Medicine and Health Care. Annu Rev Clin Psychol.

[14] Charles C, Gafni A, Whelan T (1997). Shared Decision-making in the medical encounter: what does it mean? (or It takes at least two to Tango). Soc Sci Med.

[15] Costantini M, Musso M, Viterbori P, Bonci F, Del Mastro L, Garrone O, Venturini M, Morasso G (1999). Detecting psychological distress in cancer patients: validity of the Italian version of the Hospital Anxiety and Depression Scale. Support Care Cancer.

[16] Cella DF (1997). The Functional Assessment of Cancer Therapy-Anemia (FACT-An) scale: A new tool for the assessment of outcomes in cancer anemia and fatigue. Semin Hematol.

[17] Tamburini M, Gangeri L, Brunelli C, Beltrami E, Boeri P, Borreani C, Fusco Karmann C, Greco M, Miccinesi G, Murru L, Trimigno P (2000). The hospitalised cancer patients’ needs assessment by the Needs Evaluation Questionnaire. Ann Oncol.

[18] Locke DE, Cerhan JH, Malec JF, Clark MM, Rummans TA, Brown PD (2008). Cognitive rehabilitation and problem-solving to improve quality of life of patients with primary brain tumors: a pilot study. J Support Oncol.

[19] Mainio A, Hakko H, Niemela A, Koivukangas J, Rasanen P (2006). Gender difference in relation to depression and quality of life among patients with a primary brain tumour. Eur Psychiatry.

[20] Lucchiari C, Pravettoni G, Vago G, Boiardi A (2006). Quality of life and shared decisions in patients with high grade gliomas.

[21] Zika S, Chamberlain K (1992). On the relation between meaning in life and psychological well-being. Br J Psychol.

[22] Carr AJ, Gibson B, Robinson PG (2001). Measuring quality of life: Is quality of life determined by expectations or experience. Br Med J.

[23] Salander P, Bergenheim AT, Henriksson R (2000). How was life after treatment of a malignant brain tumour. Soc Sci Med.

[24] Heimans JJ, Taphoorn MJ (2002). Impact of brain tumour treatment on quality of life. J Neurol.

[25] Grant RJ, Slattery A, Gregor P, Whittle IR (1994). Recording neurological impairment in clinical trials of glioma. J Neurooncol.

[26] Lissoni P, Cangemi P, Pirato D, Rosselli MG, Rovelli F (2001). A review on cancer-psychospiritual status interactions. Neuroendocrinol Lett.

[27] Osoba DM, Brada MD, Prados P, Yung WK (2000). Effect of disease burden on health-related quality of life in patients with malignant gliomas. Neuro Ooncol.

